# Analyzing top-down visual attention in the context of gamma oscillations: a layer- dependent network-of- networks approach

**DOI:** 10.3389/fncom.2024.1439632

**Published:** 2024-09-23

**Authors:** Tianyi Zheng, Masato Sugino, Yasuhiko Jimbo, G. Bard Ermentrout, Kiyoshi Kotani

**Affiliations:** ^1^Department of Precision Engineering, The University of Tokyo, Tokyo, Japan; ^2^Department of Mathematics, University of Pittsburgh, Pittsburgh, PA, United States; ^3^Department of Human and Engineered Environmental Studies, The University of Tokyo, Chiba, Japan

**Keywords:** visual attention, mean-field approximation, gamma oscillation, cortical column, orientation preference, quadratic integrate-and-fire model, winner-take-all

## Abstract

Top-down visual attention is a fundamental cognitive process that allows individuals to selectively attend to salient visual stimuli in the environment. Recent empirical findings have revealed that gamma oscillations participate in the modulation of visual attention. However, computational studies face challenges when analyzing the attentional process in the context of gamma oscillation due to the unstable nature of gamma oscillations and the complexity induced by the layered fashion in the visual cortex. In this study, we propose a layer-dependent network-of-networks approach to analyze such attention with gamma oscillations. The model is validated by reproducing empirical findings on orientation preference and the enhancement of neuronal response due to top-down attention. We perform parameter plane analysis to classify neuronal responses into several patterns and find that the neuronal response to sensory and attention signals was modulated by the heterogeneity of the neuronal population. Furthermore, we revealed a counter-intuitive scenario that the excitatory populations in layer 2/3 and layer 5 exhibit opposite responses to the attentional input. By modification of the original model, we confirmed layer 6 plays an indispensable role in such cases. Our findings uncover the layer-dependent dynamics in the cortical processing of visual attention and open up new possibilities for further research on layer-dependent properties in the cerebral cortex.

## 1 Introduction

Top-down attention is the ability to deliberately filter sensory information from the environment and focus on one feature out of many others. In the scope of visual attention, features like location, orientation, and object are encoded by anatomically distributed neuronal populations (Serences and Yantis, 2006). The dynamic competition of neuronal populations in the visual cortex is modulated by top-down signals from cortical areas (Katsuki and Constantinidis, 2014; Paneri and Gregoriou, 2017). Our study focuses on the selective attention of oriented bars in the receptive field, but the approach we proposed can be employed to study the dynamics of other cortical functions.

The microcircuits in the neocortex are organized in a columnar structure (Mountcastle, 1957), which is typically a six-layered architecture for each column. In the primary visual cortex, the cortical column has been found to strongly respond to bar stimuli presented in the receptive field if its orientation matches the preferred orientation of the column (Hubel and Wiesel, 1959; Reynolds et al., 1999). Moreover, if a second bar is aligned with a non-preferred orientation, the neuronal response of the column will be largely suppressed. Interestingly, if visual attention is prompted to the bar with the preferred orientation, the neuronal response of the column will recover to the original level. By comparing the level of firing rates across different sensory and attentional inputs, an ordered pattern of neuronal response can be observed (Reynolds et al., 1999; Luck et al., 1997). The mathematical analysis of the neuronal dynamics in columns is inherently challenging due to the layered structure and heterogeneity of neurons in neuronal populations (Lengler et al., 2013), even though the physiological information of connectivity between columns is available. Large-scale simulations of finite-size neuronatudy the dynamical properties of visual attention (Corchs and Deco, 2002; Wagatsuma et al., 2011; Potjans and Diesmann, 2014). However, larges-scale simulation cannot ignore the fluctuations around fixed points owing to the finite-size effect (Pikovsky and Ruffo, 1999) and random connections in the neuronal network (Landau and Sompolinsky, 2018). To analytically and systematically investigate the complex interactions between layers and populations, large-scale simulation was not enough, but mean-field approximation models in analytical form were required. In the case of heterogeneous neuronal populations, the widely used leaky integrate-and-fire (LIF) model in large-scale simulation (Wagatsuma et al., 2011; Potjans and Diesmann, 2014) cannot be dimension reduced using Lorentzian ansatz due to the leaky term. Therefore, a layer-dependent method that can intrinsically bridge single neuronal dynamics and population-level dynamics becomes indispensable.

Recent advances in neuroscience have shed light on the role of gamma oscillations mediating working memory (Pina et al., 2018), signal discrimination (Masuda and Doiron, 2007) as well as attentional processes in the visual cortex (Tiesinga and Sejnowski, 2009; Goddard et al., 2012; Bosman et al., 2012; Magazzini and Singh, 2018; Han et al., 2022). Gamma oscillations often occur in response to tonic constant current (Whittington et al., 1995; Bartos et al., 2007; Akao et al., 2018) and are thought to reflect the coordinated firing of large populations of neurons (Buzsáki and Wang, 2012; Litwin-Kumar and Doiron, 2012). Models for gamma oscillations range from Wilson-Cowan (Keeley et al., 2019) for a recent review) to very detailed models with conductance-based neurons (Traub et al., 1997). In between these two extremes, are models of individual neurons whose spiking dynamics are generated with simple first-order differential equations such as the leaky integrate-and-fire and the quadratic integrate-and-fire (QIF) models. Large networks of QIF neurons have a nice property in that under certain assumptions on the heterogeneity (e.g., parameters are taken from a Lorentzian distribution) and coupling (generally all-to-all), they can be reduced to an exact mean-field equation for each population (Montbrió et al., 2015; Dumont et al., 2017; Devalle et al., 2017; Bick et al., 2020). Thus, this intermediate level of modeling gamma oscillations allows one to carefully explore networks of networks in a computationally efficient manner.

In this paper, we start with a multi-columnar architecture including two cortical columns in the visual cortex and the neurons in populations are modeled by the QIF model. The model for mean-field neuronal dynamics is derived using the Lorentzian ansatz in order to analyze the response of the cortical columns to different sensory and attention conditions. Previous empirical studies on cats (Hubel and Wiesel, 1959) and monkeys (Luck et al., 1997; Reynolds et al., 1999; Bosman et al., 2012; Rohenkohl et al., 2018; Bogadhi et al., 2018) have demonstrated that attentional modulation can increase or decrease the firing rate or gamma-band power in the visual cortex, depending on the conditions in visual attention tasks. we reproduce these empirical findings of orientation preference and the attentional enhancement of neuronal response to validate our computational model. Then we perform parameter analysis to investigate the oscillations exhibited in the multi-columnar model and group them into several patterns. Furthermore, we investigate the modulation effect of visual attention on multiple layers and find an exceptional case that layer 2/3 and layer 5 show opposite responses to attentional input. Finally, by modifying the original multi-columnar model, we confirm that layer 6 plays an indispensable role in such layer-specific dynamics.

## 2 Materials and methods

### 2.1 Multi-columnar architecture

**Figure 1:**
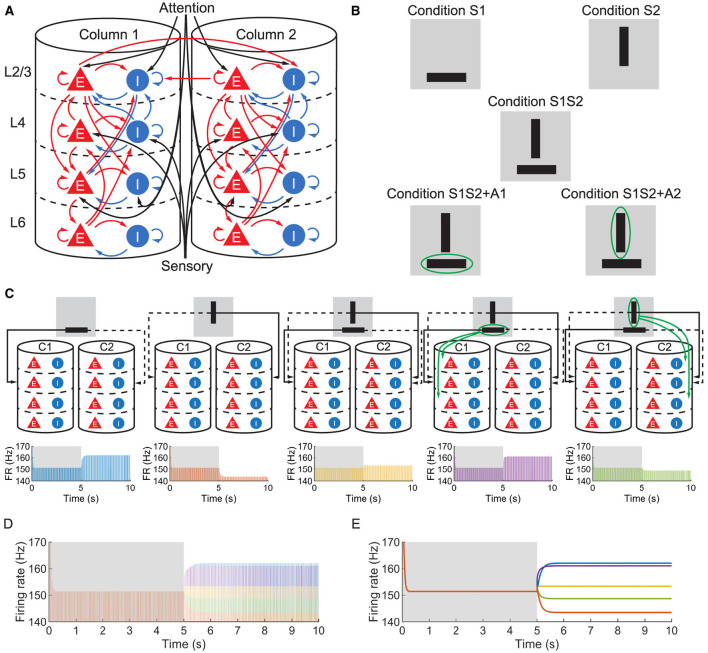
**(A)** The structure of the multi-columnar model. The red triangles with the letter E denote excitatory populations, while the blue circles with the letter I denote inhibitory populations. The pathways originating from excitatory populations are represented in red indicating an excitatory role to target populations, while the pathways originating from inhibitory populations are represented in blue indicating an inhibitory role to target populations. **(B)** Five conditions mimic sensory and attention inputs in experiments. The gray background denotes the receptive field, black bars denote visual stimuli and the green ellipse denotes attention prompting. **(C)** Time course of firing rate (FR) of population 1L5E under five conditions. Δ_*back, E*_ and Δ_*back, I*_ are set to be 0.3 and 0.02, respectively. The upper five figures are schematic diagrams of five conditions in which only sensory and attention projections are shown. The lower five figures show the firing rate of population 1L5E under five conditions respectively. Areas shaded in the gray background indicate the condition with no stimulus while areas shaded in the white background indicate conditions with sensory and/or attention input. **(D)** Time courses under five conditions are put together to compare the neuronal response to different sensory and attention inputs. The color of each time course is the same as in **(C)**. **(E)** Envelopes of time courses within the same axes as **(D)**. The color of each time course is the same as in **(C)**.

We studied a multi-columnar model of two columns in the visual cortex, depicted in Figure 1A. The structure (Wagatsuma et al., 2011; Potjans and Diesmann, 2014) and initial parameter settings (Thomson and Morris, 2002; Thomson et al., 2002; Binzegger et al., 2004) were based on previous studies. Each layer contained an excitatory population and an inhibitory population of neurons. The numbers of neurons in each population (*N*_*Y*_) were listed in Table 1. Arrows in Figure 1A represent major neuronal connections between populations. Only connections with probabilities larger than 0.04 were shown; other sparse connections were not shown. The intra-column connection probabilities of the pathway from population Y to population X $P_{Y}^{X}$ were listed in Table 2. There were also inter-column connections, projecting from the excitatory population of layer 2/3 to the inhibitory population of the same layer in the other column. The connection probability *P*_*inter*_ was set to 0.1. Neuronal populations in layer 4 of the columnar model received bottom-up sensory input, while layer 2/3 and layer 5 received top-down attention input. The projection probability of sensory and attention inputs were listed in Table 3. We mimicked visual stimulus and attention prompt of horizontal and vertical bars in the experiment as in Figure 1B. Each column had its distinct preferred orientation of bar stimuli. Neuronal populations receiving sensory input in the column were more activated if the preferred stimulus was presented and less activated with the unpreferred stimulus. The sensory input to the preferred column was ten times larger than to the unpreferred column and the sensory inputs of two bar stimuli are independent (Wagatsuma et al., 2011; Potjans and Diesmann, 2014). As the model in Figure 1A, column 1 (C1) on the left preferred the horizontal bar, and column 2 (C2) on the right preferred the vertical bar. As Figure 1B shows, five conditions are investigated in this paper, which are: S1 (only bar stimulus preferred by column 1 presented), S2 (only bar stimulus preferred by column 2 presented), S1S2 (both bar stimuli presented), S1S2 + A1 (both bar stimuli presented while the attention goes to the bar preferred by column 1), S1S2+A2 (both bar stimuli presented while the attention goes to the bar preferred by column 2).

**Table 1:** Number of neurons in each population (Wagatsuma et al., 2011).

**Population**	***N*_*Y*_**	**Population**	***N*_*Y*_**
L2/3E	10341	L2/3I	2917
L4E	10957	L4I	2739
L5E	2425	L5I	532
L6E	7197	L6I	1474

**Table 2:** Connection probabilities $P_{Y}^{X}$ (Wagatsuma et al., 2011).

		**From**							
		**L2/3E**	**L2/3I**	**L4E**	**L4I**	**L5E**	**L5I**	**L6E**	**L6I**
To	L2/3E	0.1184	0.1552	0.0846	0.0629	0.0323	0.0	0.0076	0.0
	L2/3I	0.1008	0.1371	0.0363	0.0515	0.0755	0.0	0.0042	0.0
	L4E	0.0077	0.0059	0.0519	0.1453	0.0067	0.0003	0.0453	0.0
	L4I	0.0691	0.0029	0.1093	0.1597	0.0033	0.0	0.1057	0.0
	L5E	0.1017	0.0622	0.0411	0.0057	0.0758	0.3765	0.0204	0.0
	L5I	0.0436	0.0269	0.0209	0.0022	0.0566	0.3158	0.0086	0.0
	L6E	0.0156	0.0066	0.0211	0.0166	0.0572	0.0197	0.0401	0.2252
	L6I	0.0364	0.0010	0.0034	0.0005	0.0277	0.0080	0.0658	0.1443

**Table 3:** Projection probability of sensory and attention inputs (Wagatsuma et al., 2011).

		**From**	
		**Sensory**	**Attention**
To	L2/3E	0.0	0.1
	L2/3I	0.0	0.085
	L4E	0.0983	0.0
	L4I	0.0619	0.0
	L5E	0.0	0.1
	L5I	0.0	0.085
	L6E	0.0	0.0
	L6I	0.0	0.0

### 2.2 Neuron model

The neuronal populations in the multi-columnar model were composed of Quadratic-Integrate-and-Fire (QIF) neurons (Kotani et al., 2014). The *i*-th neuron in population *X* had membrane potential *V*_*i, X*_, and was subject to the internal dynamics, synaptic current, and external input current *I*_*i, X*_, leading to


(1) $$\begin{array}{l} C\frac{dV_{i,X}}{dt}=g_{L,X}\frac{\left(V_{i,X}-V_{R}\right)\left(V_{i,X}-V_{T}\right)}{V_{T}-V_{R}}\\ -\underset{Y}{\sum }g_{Y}^{X}\left(V_{i,X}-V_{syn,Y}\right)+I_{i,X}, \end{array}$$

where *C* = 1(*μ*F/cm^2^) is the membrane capacitance, and *g*_*L, X*_ is the leak conductance, set to 0.08 (mS/cm^2^) for excitatory neurons and 0.1 (mS/cm^2^) for inhibitory neurons. *V*_*R*_ = −62 (mV) is the resting potential and *V*_*T*_ = −55 (mV) is the firing threshold. $g_{Y}^{X}$ is the synaptic conductance of the pathway from population Y to population X. *V*_*syn, Y*_ is the reversal potential, set to 0 (mV) for excitatory neurons and −70 (mV) for inhibitory neurons. *I*_*i, X*_ = *I*_*back, X*_+*I*_*sens, X*_+*I*_*attn, X*_ is the external input current, where *I*_*sens*_ is bottom-up sensory input, *I*_*attn*_ = 0.02 (mA/cm^2^) is the top-down attention input, and *I*_*back*_ is the background current. The ratio of *I*_*sens*_ and *I*_*attn*_ to excitatory and inhibitory population is determined by the projection probability listed in Table 3. The background current *I*_*back, X*_ obeys a Cauchy-Lorentzian distribution


(2) $$\begin{array}{l} f_{X}\left(I_{back,X}\right)=\frac{1}{\pi }\frac{{\text{Δ}}_{back,X}}{{\left(I_{back,X}-{Ī}_{back,X}\right)}^{2}+{\text{Δ}}_{back,X}}, \end{array}$$

where *Ī*_*back, X*_ and Δ_*back, X*_ are the center and width of the distribution, respectively. The ratio of background current to the excitatory and inhibitory population is set to be *Ī*_*back, E*_:*Ī*_*back, I*_ = 1:0.8. The dynamics of the synaptic conductance $g_{Y}^{X}$ obeys the following equation


(3) $$\begin{array}{l} \frac{dg_{Y}^{X}}{dt}=-\frac{1}{\tau_{d,Y}}g_{Y}^{X}+{ḡ}_{Y}^{X}\cdot P_{Y}^{X}\cdot \overset{N_{Y}}{\underset{k=1}{\sum }}\overset{N_{S}}{\underset{i=1}{\sum }}\delta \left(t-t_{i,Y\left(k\right)}\right), \end{array}$$

where τ_*d, Y*_ is the decay time of population Y, set to be 2 (ms) for excitatory populations and 5 (ms) for inhibitory populations (Brunel and Wang, 2003). ${ḡ}_{Y}^{X}$ is the peak conductance of the pathway from population Y to population X, and the values are listed in Table 4 to match physiologically plausible values. δ(·) is the delta function representing spikes transmitted from other neurons, and *t*_*i, Y*(*k*)_ is the time of *i*-th spike of the *k*-th neuron in the population Y. Therefore, the Equations 1–3 describe the microscopic dynamics of each neuron in the multi- columnar model.

**Table 4:** Peak conductance $g_{Y}^{X}$ (ms/cm^2^) (Brunel and Wang, 2003; Bartos et al., 2001; Gupta et al., 2000).

		**From**	
		**Excitatory population**	**Inhibitory population**
To	Excitatory population	4.069 × 10^−3^	2.672 × 10^−2^
	Inhibitory population	3.276 × 10^−3^	2.138 × 10^−2^

### 2.3 Mean-field approximation model

To study the collective behavior of neuronal populations and the interactions among populations, as well as eliminate the stochastic firing of neurons, we employ the Lorentzian ansatz (Montbrió et al., 2015) to the system and derive a mean-field approximation model for each population as follows. Firstly, to simplify the notation of the QIF model, we define ζ_*X*_, η_*X*_ and κ_*X*_ as


(4a) $$\begin{array}{ll} \zeta_{X} & =\frac{g_{L,X}}{C\left(V_{T}-V_{R}\right)}, \end{array}$$

(4b) $$\begin{array}{ll} \eta_{X} & =-\frac{g_{L,X}\left(V_{T}+V_{R}\right)}{C\left(V_{T}-V_{R}\right)}, \end{array}$$

(4c) $$\begin{array}{ll} \kappa_{X} & =\frac{g_{L,X}V_{T}V_{R}}{C\left(V_{T}-V_{R}\right)}. \end{array}$$

Applying Equation 4 to Equation 1, we get


(5) $$\begin{array}{l} {\dot{V}}_{i,X}=\zeta_{X}V_{i,X}^{2}+\eta_{X}V_{i,X}+\kappa_{X}-\underset{Y}{\sum }g_{Y}^{X}\left(V_{i,X}-V_{syn,Y}\right)+I_{i,X}. \end{array}$$

Consider the thermodynamic limit of infinite neuronal population $N_{X}\to \infty$, we drop the indices and introduce phase density function ρ_*X*_(*V*_*X*_|*I*_*X*_, *t*) to population X. At time *t*, the probability that neurons exist which membrane potential is between *V* and *V* + Δ*V* with the input current of *I*_*X*_, is $\int_{v}^{v+\text{Δ}v}\rho_{X}\left(V_{X}|I_{X},t\right)dV_{X}$. Since the number of neuron is conservative, ρ_*X*_(*V*_*X*_|*I*_*X*_, *t*) satisfies the following continuity equation


(6) $$\begin{array}{l} \frac{\partial }{\partial t}\rho_{X}\left(V_{X}|I_{X},t\right)=-\frac{\partial }{\partial V_{X}}\left[\rho_{X}\left(V_{X}|I_{X},t\right){\dot{V}}_{X}\right]. \end{array}$$

According to the Lorentzian ansatz (LA) (Montbrió et al., 2015; Akao et al., 2019), the following conditional density function in the form of Lorentzian function completely describes the low-dimensional dynamics of the neuronal population X:


(7) $$\begin{array}{l} \rho_{X}\left(V_{X}|I_{X},t\right)=\frac{f\left(I_{X}\right)}{\pi }\frac{x_{X}\left(I_{X},t\right)}{{\left[V_{X}-y_{X}\left(I_{X},t\right)\right]}^{2}+x_{X}{\left(I_{X},t\right)}^{2}}, \end{array}$$

where *x*_*X*_(*I*_*X*_, *t*) is the time-dependent half-width and *y*_*X*_(*I*_*X*_, *t*) is the center of the distribution. Together, they describe the low-dimensional dynamics of the phase density function ρ_*X*_. Apply Equation 7 to Equation 6, we obtain the low-dimensional dynamics as


(8a) $$\begin{array}{l} \frac{\partial }{\partial t}x_{X}\left(I_{X},t\right)=2\zeta_{X}x_{X}\left(I_{X},t\right)y\left(I_{X},t\right)+\eta_{X}\left(t\right)x_{X}\left(I_{X},t\right)\\ \;-\frac{1}{C}x_{X}\left(I_{X},t\right)\underset{Y}{\sum }g_{Y}^{X}, \end{array}$$

(8b) $$\begin{array}{l} \frac{\partial }{\partial t}y_{X}\left(I_{X},t\right)=-\zeta_{X}x_{X}{\left(I_{X},t\right)}^{2}+\zeta_{X}y_{X}{\left(I_{X},t\right)}^{2}+\eta_{X}\left(t\right)y_{X}\left(I_{X},t\right)\\ \;+\kappa_{X}\left(t\right)+\frac{1}{C}\left[\underset{Y}{\sum }g_{Y}^{X}V_{syn,Y}-y_{X}\left(I_{X},t\right)\underset{Y}{\sum }g_{Y}^{X}\right]+I_{X}. \end{array}$$

Consider a complex variable ω_*X*_(*I*_*X*_, *t*)≡*x*_*X*_(*I*_*X*_, *t*)+*iy*_*X*_(*I*_*X*_, *t*) and apply it to Equation 8, the dynamics of low-dimensional behavior can be combined as


(9) $$\begin{array}{l} \frac{\partial }{\partial t}\omega_{X}\left(I_{X},t\right)=i\zeta_{X}\omega_{X}{\left(I_{X},t\right)}^{2}+\eta_{X}\left(t\right)w_{X}\left(I_{X},t\right)+\kappa_{X}\left(t\right)\\ \;+\frac{1}{C}\left[\underset{Y}{\sum }g_{Y}^{X}V_{syn,Y}-\omega_{X}\left(I_{X},t\right)\underset{Y}{\sum }g_{Y}^{X}\right]+I_{X}. \end{array}$$

Now we introduce two collective observables: firing rate *r*_*X*_(*t*) and mean membrane potential *v*_*X*_. The firing rate *r*_*X*_(*t*) can be derived by integrating the flow velocity in the dynamics of the phase density function for all *I*_*X*_ at the point *V*_*X*_ = *V*_*peak*_, where *V*_*peak*_ is the firing threshold. If we set *V*_*peak*_ approaches infinity as $V_{peak}\to \infty$, *r*_*X*_(*I, t*) can be computed by


(10) $$\begin{array}{l} r_{X}\left(I,t\right)=\rho_{X}\left(V_{X}\to \infty |I_{X},t\right){\dot{V}}_{X}\left(V_{X}\to \infty |I_{X},t\right). \end{array}$$

Apply Equations 5, 7 to Equation 10, one can obtain the simple identity


(11) $$\begin{array}{l} r_{X}\left(I,t\right)=\frac{f\left(I_{X}\right)}{\pi }x_{X}\left(I_{X},t\right). \end{array}$$

The total firing rate *r*(*t*) comes to be


(12) $$\begin{array}{l} r_{X}\left(t\right)=\frac{1}{\pi }\int_{-\infty }^{\infty }x_{X}\left(I_{X},t\right)f\left(I_{X}\right)dI_{X}. \end{array}$$

On the other hand, *y*_*X*_(*I*_*X*_, *t*) is the mean of the membrane potential for each *I*_*X*_ value:


(13) $$\begin{array}{l} y_{X}\left(I_{X},t\right)=\text{p.v.}\int_{-\infty }^{\infty }\rho_{X}\left(V_{X}|I_{X},t\right)V_{X}dV_{X}. \end{array}$$

Note that this integral is defined by the Cauchy principal value $\text{p.v.}\int_{-\infty }^{\infty }h\left(x\right)dx=\lim_{R\to \infty }\int_{-R}^{R}h\left(x\right)dx$, in order to eliminate the uncertainty of the integral. Then mean membrane potential is then


(14) $$\begin{array}{l} v_{X}\left(t\right)=\int_{-\infty }^{\infty }y_{X}\left(I_{X},t\right)f_{X}\left(I_{X}\right)dI_{X}. \end{array}$$

Since *I*_*X*_ = *I*_*back, X*_ + *I*_*sens, X*_ + *I*_*attn, X*_ and *I*_*back, X*_ follows a Lorentzian distribution, as Equation 2, the integrals in Equations 12, 14 can be analytically evaluated over the closing contour in the complex *I*_*X*_ plane using the residue theorem. Thus the firing rate *r*_*X*_ and mean membrane potential *v*_*X*_ are determined by the value of ω at the pole of *f*(*I*_*X*_) in the lower half *I*_*X*_ plane:


(15) $$\begin{array}{l} \pi r_{X}\left(t\right)+iv_{X}\left(t\right)=\omega \left({Ī}_{X}-i{\text{Δ}}_{X},t\right) \end{array}$$

**Figure 2:**
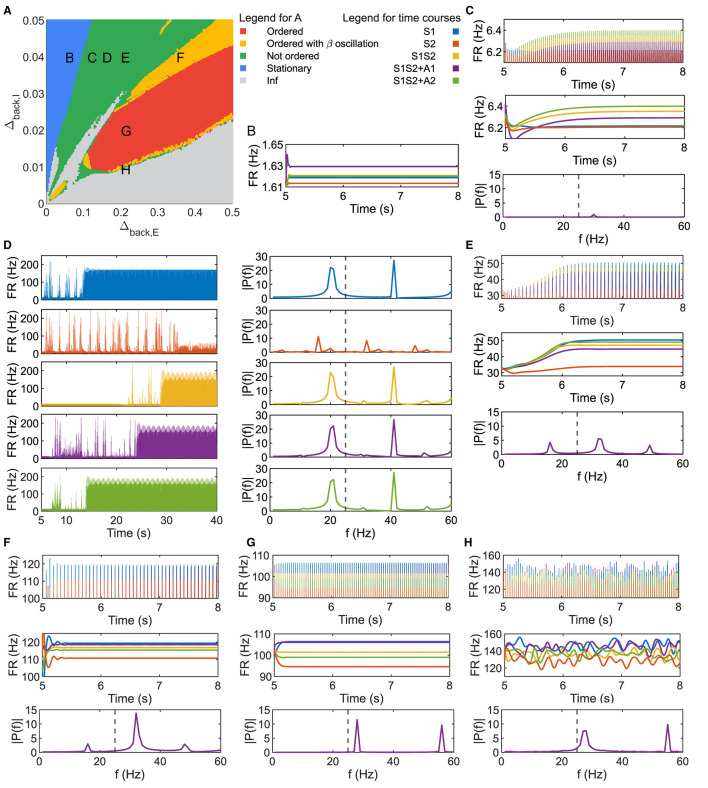
Parameter plane analysis shows different patterns of neuronal response. **(A)** Parameter plane of Δ_*back, E*_ and Δ_*back, I*_. **(B–H)** Firing rate of the population 1L5E with parameter settings corresponding to the location of letters marked on **(A)**. **(B)** only shows the raw data of the firing rate. **(D)** shows the raw data and power spectrum of the firing rate under each condition respectively. **(C, E–H)** show raw data, envelope, and power spectrum of firing rate where the power spectrums are for condition S1S2 + A1. Dashed lines in the amplitude spectra denote *f* = 25 (Hz). Parameter setting of the picked points: (B, Δ_*back, E*_ = 0.05, Δ_*back, I*_ = 0.04); (C, Δ_*back, E*_ = 0.11, Δ_*back, I*_ = 0.04); (D, Δ_*back, E*_ = 0.15, Δ_*back, I*_ = 0.04); (E, Δ_*back, E*_ = 0.2, Δ_*back, I*_ = 0.04); (F, Δ_*back, E*_ = 0.35, Δ_*back, I*_ = 0.04); (G, Δ_*back, E*_ = 0.2, Δ_*back, I*_ = 0.02); (H, Δ_*back, E*_ = 0.2, Δ_*back, I*_ = 0.0095).

Finally, we evaluate Equation 9 at *I*_*X*_ = *Ī*_*back, X*_+*I*_*sens, X*_+*I*_*attn, X*_−*iΔ*_*back, X*_, and thus obtain Equation 16 as


(16a) $$\begin{array}{ll} \frac{dr_{X}}{dt} & =2\zeta_{X}r_{X}v_{X}+\eta_{X}r_{X}-\frac{r_{X}}{C}\underset{Y}{\sum }g_{Y}^{X}+\frac{\zeta_{X}}{\pi }{\text{Δ}}_{back,X}, \end{array}$$

(16b) $$\begin{array}{l} \frac{dv_{X}}{dt}=\zeta_{X}v_{X}^{2}+\eta_{X}v_{X}+\kappa_{X}-\frac{\pi^{2}}{\zeta_{X}}r_{X}^{2}\\ \;+\frac{1}{C}\left(\underset{Y}{\sum }g_{Y}^{X}V_{syn,Y}-v_{X}\underset{Y}{\sum }g_{Y}^{X}\right)\\ \;+{Ī}_{back,X}+I_{sens,X}+I_{attn,X}, \end{array}$$

(16c) $$\begin{array}{ll} \frac{dg_{Y}^{X}}{dt} & =-\frac{1}{\tau_{d,Y}}g_{Y}^{X}+{ḡ}_{Y}^{X}\cdot P_{Y}^{X}\cdot N_{Y}\cdot r_{Y}, \end{array}$$

where *r*_*X*_ and *v*_*x*_ are the firing rate and mean membrane potential of population X. The ratio of three current sources is set to be *I*_*sens*_:*I*_*attn*_:*Ī*_*back*_ = 9:3:16 (Wagatsuma et al., 2011). Therefore, the Equation 16 described the mean-field dynamics of each population in the multi-columnar model. The simulations in this paper were all based on Equations 4, 16. All the simulations are integrated by the Euler method, with a time step Δ*t* = 0.01(ms), using MATLAB (R2022b, https://www.mathworks.com/products/matlab.html). For simulations except for Figure 2D, we simulated 10 s of neuronal activity. The MATLAB code for simulation and analysis is available at https://github.com/Shawnzty/multicolumn.

## 3 Results

To confirm the validity of our multi-columnar model composed of the neuronal population with mean-field approximation, we first reproduced the empirical findings about orientation preference and the enhancement of neuronal response due to visual attention. In order to validate our model, which consists of a neuronal population with mean-field approximation, we first sought to replicate the observed phenomena of orientation preference and the amplification of neuronal response due to visual attention.

### 3.1 The firing rate of the excitatory population in layer 5 shows an ordered pattern

Layer 5 of the visual cortex was found to be an integration center for the signal from all other layers of the cortical column and project the integrated signal to other regions of the neocortex or subcortical structures (Kasper et al., 1994; Tang and Higley, 2020; Shai et al., 2015). Therefore, we focused on the firing rate of the excitatory population in layer 5 of column 1 (hereafter referred to as 1L5E). For all conditions, both with and without sensory and attention input, the population 1L5E generates gamma oscillations whose center frequency is 30 (Hz) (Figure 1C). Note that we simulated the system for 5 s before applying sensory and/or attention input, so the influence of initial conditions on the dynamics could be eliminated. In Figure 1D, we aligned all the time courses in the same axes and found that the center frequency didn't change with the condition of sensory and attention input. Only the amplitudes of oscillation were changed due to sensory and attention input. For condition S1, since the sensory input was preferential to column 1, 1L5E was activated and the amplitude of oscillation reached a higher level than the steady state. For condition S2, the sensory input was preferential to column 2, so the sensory input to column 1 was ten times less than condition S1; because of the inhibition from column 2 to column 1, the amplitude of oscillation decreased to a lower level than the steady state. When both sensory inputs were presented (condition S1S2), due to the inhibition from column 2 to column 1, the amplitude of oscillation reached a medium level, suppressed from condition S1. However, if the attention input was prompted to the preferred stimulus (condition S1S2 + A1), the amplitude of oscillation was increased to a higher level than condition S1S2. Finally, the attention prompted to column 2 increased the inhibition from column 2 to column 1 resulting in a lower level than the condition S1S2. To compare the amplitude of oscillations, we aligned the envelope of firing rate under five conditions in the same axes (Figure 1E) and found an ordered pattern of firing rates:


S1, S1S2 + A1>S1S2>S1S2 + A2, S2.

This ordered pattern of the amplitude of firing rates agreed with empirical findings on cat (Hubel and Wiesel, 1959), primate (Luck et al., 1997; Reynolds et al., 1999; Bosman et al., 2012; Rohenkohl et al., 2018; Bogadhi et al., 2018) and other computational simulations (Wagatsuma et al., 2011; Potjans and Diesmann, 2014).

### 3.2 The ordered pattern only appears in a restricted region in the parameter plane of heterogeneity

The heterogeneity of neurons played a pivotal role in influencing characteristics of gamma oscillations (Wang and Buzsáki, 1996; Litwin-Kumar and Doiron, 2012; So et al., 2014) and attention (Zdorovtsova et al., 2023; Daitch and Parvizi, 2018). Notably, studies have shown that the dispersion of background input currents directly shaped neuronal heterogeneity even within the same type (Montbrió et al., 2015; Zheng et al., 2021). In our study, we manipulated the dispersion of background current in our model by two parameters Δ_*back, E*_ and Δ_*back, I*_, which denote heterogeneity of excitatory and inhibitory populations respectively. In light of the observed ordered pattern, we investigate the potential relationship between its appearance and the heterogeneity of background input current. Of the many possible parameters to vary, we chose the dispersion of the background inputs (heterogeneity) as we found that these are a major factor in determining the existence of gamma oscillations (see, e.g. Figure 4 of Devalle et al., 2017).

Figure 2A is the parameter plane of (Δ_*back, E*_, Δ_*back, I*_) divided by five colored regions. In the blue region, the heterogeneity of background current for the inhibitory populations was relatively large while heterogeneity for the excitatory populations was relatively small on the parameter plane, resulting in the inhibition being strong and no oscillations were observed, as Figure 2B. Since the system usually took < 2 s to reach the new steady state under sensory and/or attention input, we showed the dynamics up to 3 s after applying external input (t = 5 – 8(s)) in the figures hereafter.

While oscillations in neuronal response were observed in the green region, the firing rate in Figures 2C–2E, H showed that four patterns of the amplitudes were possible with sensitive parameter selection and none of them were in same ordered pattern as Figures 1D, E. The amplitude spectrum in the bottom panel of Figure 2C was obtained by performing a Fourier transform on the firing rate data under condition S1S2 + A1 in the last second, the same as all the amplitude spectra later. The only peak of the frequency amplitude was in the gamma band and the amplitude in the slower frequency band was always zero. The parameter setting at letter D on the plane showed nontrivial activities as the raw firing rate in Figure 2D. For all conditions, the population did not immediately reach a new steady state, but took a very long time. The power spectrum showed different patterns of firing rate at the new steady state under sensory and/or attentional input. Besides, we observed that the firing rate at the new steady state contained both beta and gamma frequency bands. Figure 2E showed another amplitude pattern with beta and gamma frequency bands, with two peaks in the gamma band. Note that the parameter setting of the letter H was in the green region beside the boundary of multiple regions. Figure 2H showed that the neuronal responses kept exhibiting fluctuations at the level of the original steady state. The dominant frequency was in the gamma band but the component in the beta band was non-zero.

The parameter setting in the red region can reproduce an ordered pattern similar to the one in Figure 1E. The amplitude spectrum in Figure 2G shows two peaks of frequency amplitude in the gamma band and no amplitude in lower frequency bands. In the yellow region, as Figure 2F showed, the order of oscillation amplitude was consistent with the ordered pattern as Figures 1E, 2G, while the beta frequency exists in the firing rate.

### 3.3 Layers 2/3 and 5 can exhibit opposite responses to attentional input

Several models of neuronal networks have been proposed to elucidate the underlying mechanisms of selective activation and attentional enhancement in the visual cortex (Reynolds et al., 1999; Boynton, 2005; Buia and Tiesinga, 2008). The winner-take-all (WTA) theory, particularly, has been instrumental in capturing the dynamic competition between columns (Fukai and Tanaka, 1997; Wagatsuma et al., 2011). The WTA theory can be summarized as when inter-column inhibition dominates over intra-column inhibition, a unique winner survives in the competition. Large-scale simulation of neuronal networks confirmed the winner-take-all dynamics in the attention selection of stimulus competition mediated by gamma oscillations (Börgers et al., 2008). According to the previous study based on similar settings (Wagatsuma et al., 2011), the WTA of the multi-columnar model happened in layer 2/3, where the firing rate of the excitatory population in layer 2/3 (hereafter refer to the one in column 1 as 1L2/3E) exhibits characteristic dynamics. As can be seen in Figure 1A, in our model, inter-column interactions are mediated only by layer 2/3, so it is expected that if WTA is necessary for an ordered pattern, we should not be able to get the ordered pattern if WTA across layer 2/3 does not hold.

**Figure 3:**
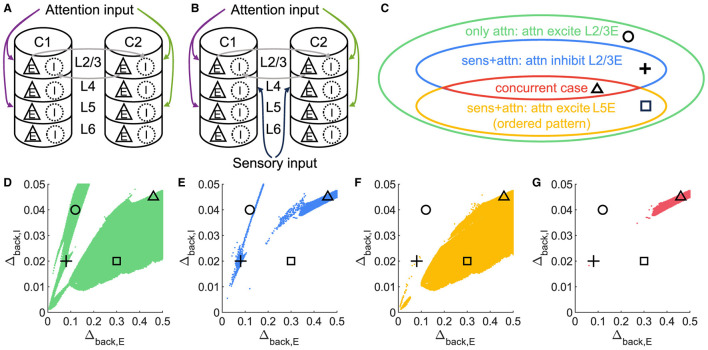
A concurrent case that 1L2/3E and 1L5E exhibit opposite responses to attentional input. **(A, B)** Multi-columnar model under conditions with only attention input **(A)** or with both sensory and attention input **(B)**. All connections among neuronal populations are not shown. **(C)** Venn diagram shows the relation of various state sets. Circle, plus, triangle, and square markers represent typical parameter settings that result in four cases. **(D–G)** Parameter planes of Δ_*back, E*_ and Δ_*back, I*_. The colors of scatter plots are consistent with those used in **(C)**. Parameter setting of the picked points: (Circle, Δ_*back, E*_ = 0.12, Δ_*back, I*_ = 0.04); (Plus, Δ_*back, E*_ = 0.08, Δ_*back, I*_ = 0.02); (Square, Δ_*back, E*_ = 0.3, Δ_*back, I*_ = 0.02); (Triangle, Δ_*back, E*_ = 0.46, Δ_*back, I*_ = 0.045).

**Figure 4:**
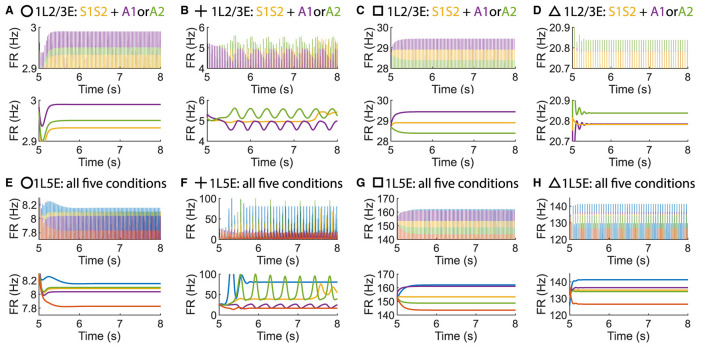
Time courses of firing rate of population 1L2/3E and 1L5E with parameter settings represented by markers in Figures 3D–G. **(A, E)** corresponds to the parameter setting at the circle marker. **(B, F)** corresponds to the parameter setting at the plus marker. **(C, G)** corresponds to the parameter setting at the square marker. **(D, H)** corresponds to the parameter setting at the triangle marker. The upper and lower panels of figures are raw data and the envelope of firing rate, respectively. Line colors correspond to five conditions in Figure 1C.

Surprisingly, it is possible to maintain an ordered pattern in layer 5 and get anti-WTA behavior in layer 2/3 (that is, *S1S2+A2*>*S1S2+A1*). Figure 3 depicts various behaviors of layer 2/3 in the parameter plane under 2 scenarios: A attention only and A attention plus stimulus to both columns (S1S2) (see Figures 3A, B). In the green regions of the parameter plane (Figure 3D), attention in column 1 leads to increased activity in column 1 and decreased activity in column 2 because the excitation in the attended column engages the inhibition in the unattended column. The yellow region in Figure 3F corresponds to parameters that show the normal ordered pattern in layer 5. When equal sensory inputs are given to both columns and attention is given to column 1, then we expect that the layer 2/3 excitatory activity in column 1 will exceed that of column 2 because of the WTA property. However, as seen in Figure 3E, there is a small blue region of parameter space where S1S2 + A1 reduces the excitatory activity in 1L2/3E while S1S2+A2 enhances it. There is a small intersection of this area with that in Figure 3E, shown in Figure 3G where the normal response to attention occurs in layer 5 but anti-WTA takes place in layer 2/3 (labeled the concurrent case). Subfigures in the top row of Figure 4 show the responses of excitatory cells in column 1 layer 2/3 under the S1S2 and S1S2 + A1 or A2 stimulus conditions at each of the four points shown in the parameter planes in Figures 3D–G. The bottom row shows the behavior of layer 5 in all five conditions. Recall that the normally ordered pattern in layer 5 is


S1, S1S2 + A1>S1S2>S1S2 + A2, S2.

In particular, Figures 4D, H show that in the S1S2 + A1 condition excitatory activity increases in layer 5 column 1 (H) but decreases in layer 2/3 (D). That is, WTA in layer 2/3 is not necessary for an ordered pattern in layer 5 and, in fact, the ordered pattern occurs even if layer 2/3 shows anti-WTA behavior.

### 3.4 Layer 6 is crucial in the opposite responses of layers 2/3 and 5 to attentional input

**Figure 5:**
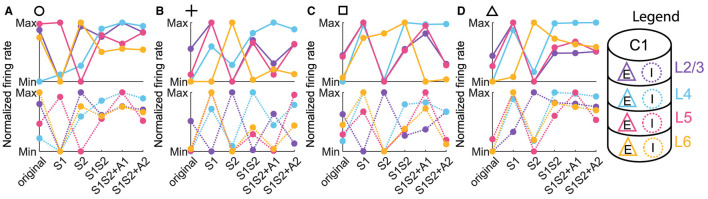
Normalized firing rate across input conditions for each neuronal population. **(A–D)** The normalized firing rate of excitatory (upper panels) and inhibitory (lower panels) populations in column 1 with parameter settings represented by markers in Figures 3D–G. Line and dot colors denote layers in column 1, and the solid or dot line denotes the excitatory or inhibitory population, corresponding to those in the legend.

To get some understanding of the concurrent scenario, in Figure 5, we look at the normalized excitatory and inhibitory firing rates in each of the 4 layers across the six conditions. In particular, we focus on Figures 5C, D corresponding to the normal ordered pattern and the concurrent cases from Figures 3F, G. For each neuronal population, we normalized the firing rate value by using the maximum and minimum values across all six conditions, including the activity before the stimulus and five types of stimulus.

Three observations can be extracted from the line chart of the normalized firing rate.

1. It was evident that the response trends of both excitatory and inhibitory populations vary considerably across layers. This implied a layer-dependent modulation of neuronal excitability, rejecting the notion that the column of neuronal clusters operates as a single, uniform entity. Rather, our observations suggested a more nuanced interplay of activity, where different layers exhibited unique responses to distinct sensory and attention conditions.
2. We discovered that the response trends of excitatory and inhibitory populations were not necessarily in the same direction or opposite. Specifically, an increase in the firing rate of the excitatory population due to a change in condition did not unambiguously predict the behavior of the inhibitory population, which can be either more activated or inhibited.
3. Since we are interested in the concurrent case that 1L2/3E and 1L5E exhibiting opposite responses to attentional input, we focused on Figures 5C, D. We observed that the excitatory population in Layer 6 (1L6E, represented by the solid yellow line in the upper panels) exhibits inverse changes from S1S2 + A1 to S1S2+A2 in parameter settings indicated by square and triangle markers. This observation suggests the possibility that layer 6 may influence the occurrence of the concurrent case.

**Figure 6:**
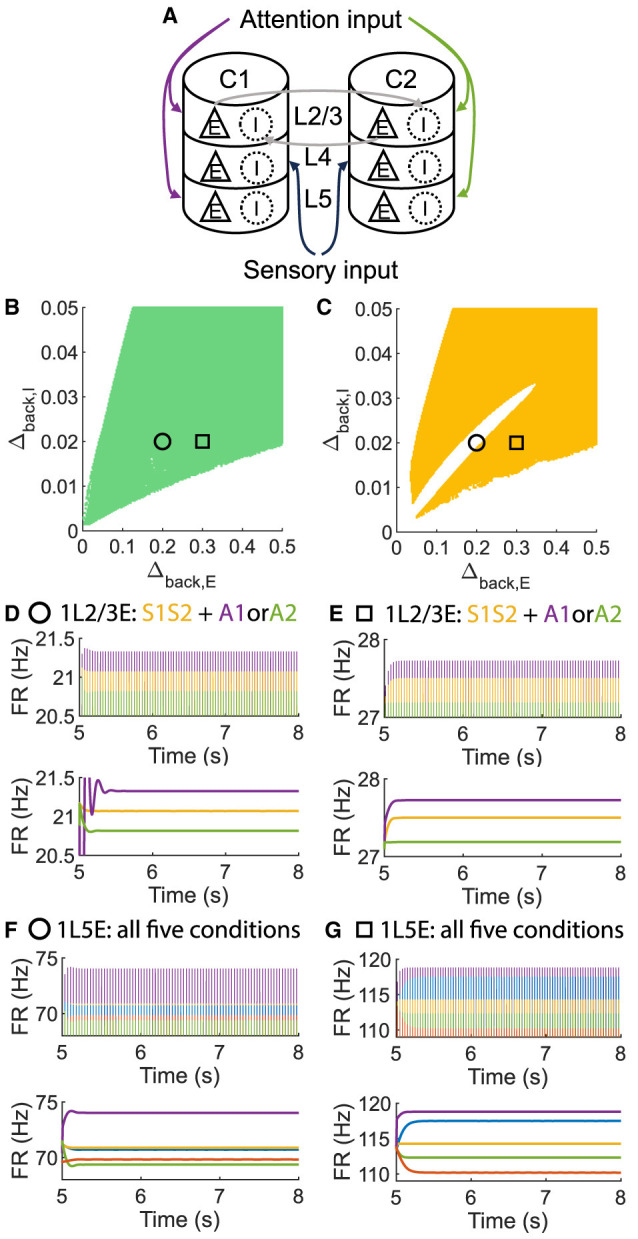
The ordered pattern exists when layer 6 is removed in the multi-columnar model, but neither the anti-WTA case nor the concurrent case exists. **(A)** Multi-columnar model with layer 6 removed. **(B, C)** Parameter plane of Δ_*back, E*_ and Δ_*back, I*_. The colors of scatter plots are consistent with those used in Figures 3D–G. **(D–G)** Time courses of firing rate of population 1L2/3E and 1L5E with parameter settings represented by markers in **(B, C)**. The upper and lower panels of subfigures are raw data and the envelope of firing rate, respectively. Line colors correspond to five conditions in Figure 1C. Parameter setting of the picked points: (Circle, Δ_*back, E*_ = 0.2, Δ_*back, I*_ = 0.02); (Square, Δ_*back, E*_ = 0.3, Δ_*back, I*_ = 0.02).

**Figure 7:**
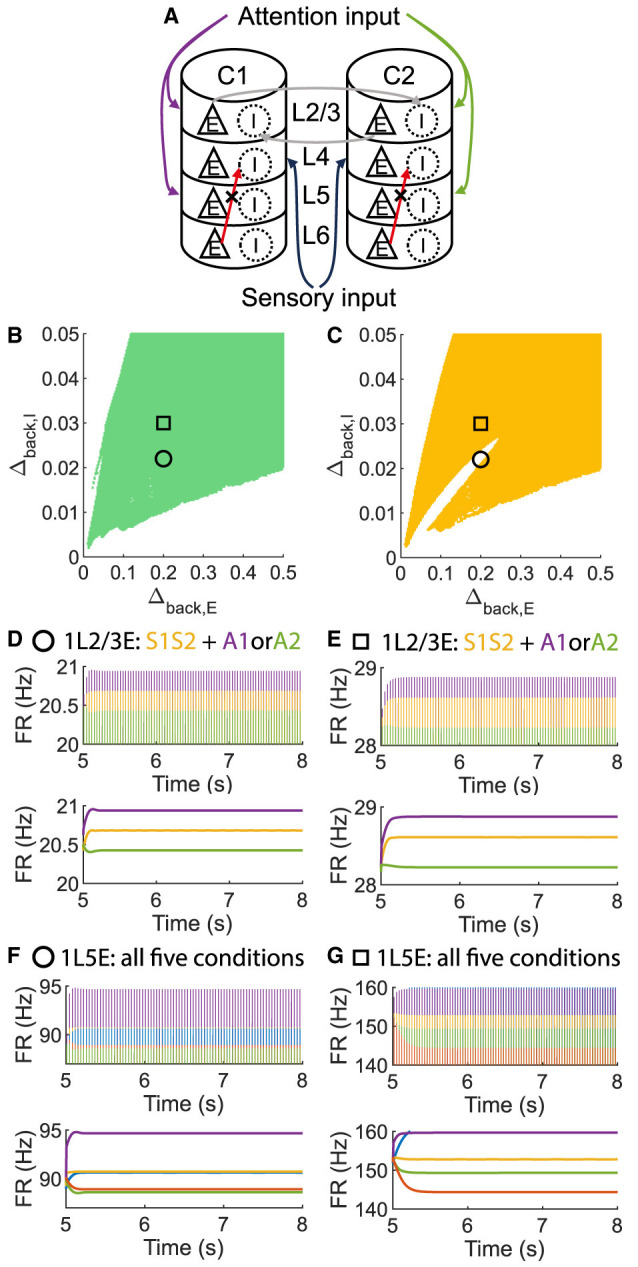
The ordered pattern exists when the pathway from L6E to L4I is blocked in the multi-columnar model, but neither the anti-WTA case nor the concurrent case exists. **(A)** Multi-columnar model with the pathway from L6E to L4I blocked. **(B, C)** Parameter plane of Δ_*back, E*_ and Δ_*back, I*_. The colors of scatter plots are consistent with those used in Figures 3D–G. **(D–G)** Time courses of firing rate of population 1L2/3E and 1L5E with parameter settings represented by markers in **(B, C)**. The upper and lower panels of subfigures are raw data and the envelope of firing rate, respectively. Line colors correspond to five conditions in Figure 1C. Parameter setting of the picked points: (Circle, Δ_*back, E*_ = 0.2, Δ_*back, I*_ = 0.022); (Square, Δ_*back, E*_ = 0.2, Δ_*back, I*_ = 0.03).

**Figure 8:**
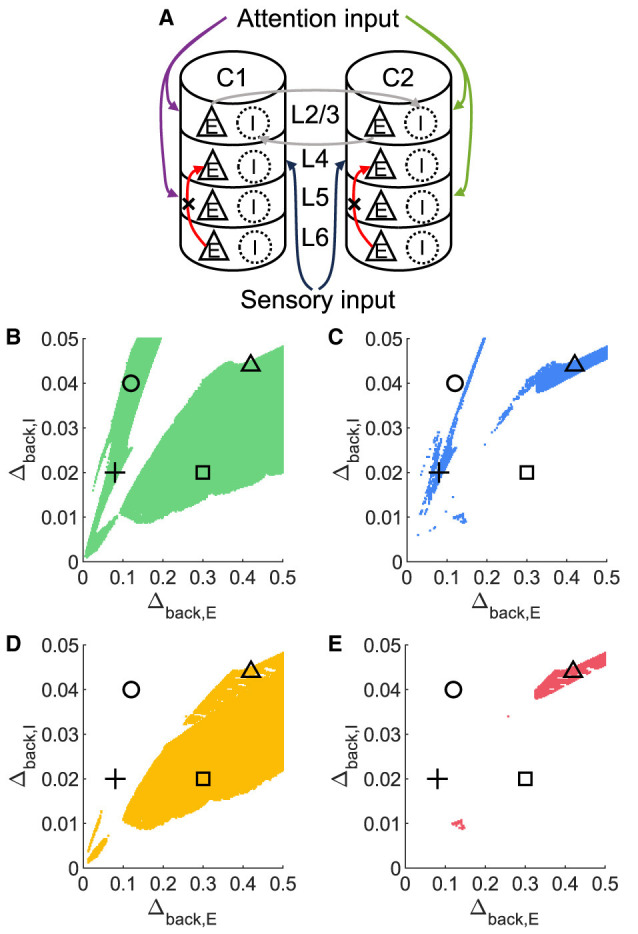
The concurrent case exists when the pathway from L6E to L4E is blocked in the multi-columnar model. **(A)** Multi-columnar model with the pathway from L6E to L4E blocked. **(B–E)** Parameter plane of Δ_*back, E*_ and Δ_*back, I*_. The colors of scatter plots are consistent with those used in Figures 3D–G. Parameter setting of the picked points: (Circle, Δ_*back, E*_ = 0.12, Δ_*back, I*_ = 0.04); (Plus, Δ_*back, E*_ = 0.08, Δ_*back, I*_ = 0.02); (Square, Δ_*back, E*_ = 0.3, Δ_*back, I*_ = 0.02); (Triangle, Δ_*back, E*_ = 0.42, Δ_*back, I*_ = 0.044).

**Figure 9:**
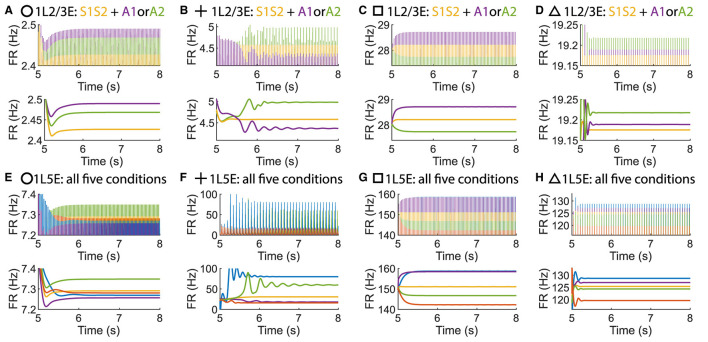
Time courses of firing rate of population 1L2/3E and 1L5E with parameter settings represented by markers in Figures Figures 8B–E. **(A, E)** corresponds to the parameter setting at the circle marker. **(B, F)** corresponds to the parameter setting at the plus marker. **(C, G)** corresponds to the parameter setting at the square marker. **(D, H)** corresponds to the parameter setting at the triangle marker. The upper and lower panels of figures are raw data and the envelope of firing rate, respectively. Line colors correspond to five conditions in Figure 1C.

To investigate whether layer 6 was necessary for the concurrent case, we adjusted the architecture outlined in Figure 1A by removing layer 6 from the multi-columnar model (Figure 6A). To achieve so, we set the connection probabilities between layer 6 and other layers to be zero, while keep all the other parameters the same as the original setting in Table 2. We confirmed the existence of the ordered pattern in the revised model, as the region shaded in yellow in Figure 6C. A typical time course of 1L5E presenting an ordered pattern was shown in Figure 6G. However, we found neither anti-WTA dynamics nor concurrent case in the revised model and the responses of 1L2/3 and 1L5E to attentional input were always in the same direction within the ordered pattern scenario (Figures 6D–G). One possible reason for this can be seen by referring to the pathways in Figure 1A. The heightened level of excitatory activity in layer 6 during S1S2 + A1 increases the inhibitory activity in layer 4 (compare Figures 5C, D) which projects directly to layer 2/3 excitatory neurons and lowers their activity. To test this hypothesis, we blocked the pathway from L6E to L4I in the original model and simulated the modified model (Figure 7A). To achieve so, we set the connection probabilities between these two populations to be zero ($P_{L6E}^{L4I}=0$), while keep all the other parameters the same as the original setting in Table 2. Similar to the result obtained in the layer 6 removed model in Figure 6, we found the ordered pattern on the parameter plane but both the anti-WTA case and concurrent case disappeared (Figures 7B, C). The responses of 1L2/3 and 1L5E to attentional input were always in the same direction within the ordered pattern scenario (Figures 7D–G). Since layer 6 only has strong projections to excitatory and inhibitory populations in layer 4 (Figure 1A), we also tested the existence of concurrent cases with pathway L6E to L4E blocked (Figure 8A). To achieve so, we set the connection probabilities between these two populations to be zero ($P_{L6E}^{L4E}=0$), while keep all the other parameters the same as the original setting in Table 2. We found a region of anti-WTA dynamics (Figure 8C), a region of the ordered pattern (Figure 8D), and further an interaction region of the concurrent case (Figure 8E) when pathway from L6E to L4E was blocked. The attention input can either increase (Figure 9A) or decrease (Figure 9B) the firing rate of the population L2/3E depends on the parameter settings. The ordered pattern only appears in a restricted region, but not on the entire plane (Figures 9E, F). What's more, the firing rate of 1L2/3E can achieve a higher level either with an attentional input (Figure 9C) or without an attentional input (Figure 9D), while 1L5E maintained an ordered pattern of firing rate (Figures 9G, H). Therefore, we confirmed the indispensable role of the pathway from L6E to L4I for the occurrence of the concurrent case.

## 4 Discussion

### 4.1 Model significance and comparison with previous models

Columns in the visual cortex have an intrinsic preference for oriented bars of specific orientation when presented in their receptive fields; multiple oriented bars induce competition between neighboring columns (Hubel and Wiesel, 1959; Reynolds et al., 1999). In order to explore this in the context of gamma oscillations, we have developed a multi-layer model with mean-field approximated neuronal populations for each cortical column in the visual cortex.

The network-of-networks feature of cortical columns presents difficulties for computational studies, especially in the context of gamma oscillations. Large-scale simulation of neuronal populations cannot avoid the finite-size effects, and the unstable nature of gamma oscillations causes stochastic dynamics in simulation. We referred the parameter settings of structure and connectivity of previous large-scale simulation studies (Wagatsuma et al., 2011; Potjans and Diesmann, 2014), but the proposed model were different in single neuron model, heterogeneity in neuronal populations and mean-field approximation. The referred studies studied LIF model with identical neurons while the current study considered QIF model with heterogeneous neurons. This is due to the need of performing mean-field approximation to neuronal populations and further systematically investigate the attentional modulation under the changing parameters.

We proposed a layer-dependent network-of-networks approach with experimentally obtained connectivity. The mesoscopic model for each heterogeneous neuronal population, as Equation 16, is derived from the QIF model using Lorentzian ansatz. The mean-field approximation of identical neuronal populations composed of leaky integrate-and-fire (LIF) neurons was explored in previous studies (Brunel, 2000; Schwalger et al., 2017). However, the Lorentzian ansatz used to reduce the dimensionality of the heterogeneous population cannot be employed in the LIF model due to the shape of the leaky term. We extended the LIF model, based on linear transfer function, to the QIF model and employed Lorantzian ansatz to capture the collective dynamics of heterogeneous neuronal populations. The dynamics of each population is described by three equations: firing rate *r*_*X*_, mean membrane potential *v*_*X*_, and synaptic conductance $g_{Y}^{X}$. The entire multi-columnar system consists of 16 populations including two columns, four layers for each column, and two populations in each layer. The derived mesoscale model enables us to investigate dynamics with gamma oscillation which cannot be achieved by conventional reduced models such as Wilson Cowan model (Wilson and Cowan, 1972). It is known that the firing of neurons in the visual cortex representing orientation preference is associated with gamma oscillation (Bosman et al., 2012; Han et al., 2022). The derived model allows us to investigate the relationship between orientation preference with attentional enhancement and the gamma oscillations in a layer-dependent manner.

### 4.2 Heterogeneity of neuronal population in the context of gamma oscillation

Rather than assume homogeneous neuronal populations, since the heterogeneity among the population strongly affects the characteristics of gamma oscillations (Wang and Buzsáki, 1996; Litwin-Kumar and Doiron, 2012; So et al., 2014) and attention function (Zdorovtsova et al., 2023; Daitch and Parvizi, 2018), we manipulated the distribution dispersion of background current *I*_*back*_ in the same type of neurons, which results in diverse activity of neurons in each population. As the parameter plane (Figure 2A) represents, the system exhibited a stationary state (blue region) with large Δ_*back, I*_ (inhibitory background current dispersion) and small Δ_*back, E*_ (excitatory background current dispersion). With this level of heterogeneity, the network is unable to synchronize into a coherent rhythm, similar to the behavior of the Kuramoto model with large natural frequency dispersion (Kuramoto, 1984). Heterogeneity of inhibitory neurons has been previously shown to induce asynchronous behavior and suppress oscillations (Wang and Buzsáki, 1996; Pazó and Montbrió, 2014; Zheng et al., 2021). The situation is more complex for excitatory-inhibitory systems because the coupling between the excitatory units and inhibitory units as well as the topology of the network impacts the synchronization (Montbrió and Pazó, 2018). Here, we found a wide region in the heterogeneity parameter plane where the ordered pattern of visual attention was in the context of gamma oscillations. We showed that gamma oscillations help the attentional control of orientation selectivity (Magazzini and Singh, 2018), which complements and provides new insights to the previous computational results obtained by the numerical simulation of large-scale neuronal populations (Wagatsuma et al., 2011; Potjans and Diesmann, 2014). In addition, we found not only gamma oscillations but also slower (beta frequency) oscillations and an aperiodic oscillatory state in a considerable parameter region of Δ_*back, I*_ and Δ_*back, E*_, as shown in Figure 2A. Such results were consistent with a recent study that found mixed gamma and beta/alpha rhythms across cortical layers in a gradient motif (Mendoza-Halliday et al., 2024), and a previous study of network models (i.e. couplings of clustered populations) (Litwin-Kumar and Doiron, 2012).

In Litwin-Kumar et al. (2016), the authors modeled an inhibitory/excitatory network and introduced heterogeneity among the inhibitory cells by dividing them into heterogeneous subtypes: parvalbumin (PV), somatostatin (SOM), and vasointestinal peptide-expressing (VIP) interneurons. The subtypes were distinguished by properties such as threshold, adaptation, connectivity, and rise-time of the synapses. Their work reproduced and predicted the roles of different subtypes in several phenomena including disinhibition, surround suppression, and modulation of orientation tuning all in the context of steady-state responses. The heterogeneity in our networks arises from the driving current; in a sense, the equivalent of setting different firing thresholds for our neurons. Although our work considered only a unimodal distribution for *I*_*back*_, the region of the ordered pattern became wider with the increase of Δ_*back, I*_ (Figure 2A), demonstrating the importance of the diversity in the characteristics of interneurons for signal discrimination in the visual cortex. The same Ott-Antonsen approach that we have used here could be employed to study other aspects of heterogeneity on the firing patterns and rhythms of neurons in cortical networks (Gast et al., 2024) as well as the possibility of adding other neuronal subtypes.

### 4.3 The ordered pattern induced by visual attention beyond WTA theory

Previous studies provided a winner-take-all theory to explain the attentional selection in the visual cortex (Fukai and Tanaka, 1997; Wagatsuma et al., 2011; Chen, 2017; Lee et al., 1999; Zénon et al., 2009). While this theory provided a substantial framework, it overlooked the intricacies of network structures and homogenized the layer-specific inputs and connectivity. Instead, we took the internal structure of the cortical column into consideration so that it was possible to investigate the winner-take-all theory in a more detailed manner. The number of neurons in each population and the connection between layers were determined by experimental findings. Furthermore, our model employed layer-specific input and fixed connectivity (Table 2) between populations. Thus our quantitative and layer-dependent approach found a concurrent case that kept an ordered pattern of layer 5 (Figure 4H) while the dynamics of layer 2/3 did not conform to WTA theory (Figure 4D). The emergence of opposite responses of 1L2/3E and 1L5E in the presence of sensory input suggested a complex interplay beyond WTA dynamics.

Our analysis revealed the response of excitatory and inhibitory populations to the sensory and attention input varied considerably across layers. This finding demonstrated that neuronal clusters in a column did not operate uniformly but exhibited distinct response profiles in different layers, such as were reported in animal electrophysiology and human fMRI studies (Senzai et al., 2019; Olman et al., 2012). The network-of-networks approach proposed in this paper is also a promising method beyond the visual cortex. Since layer-specific features were also found in rodent auditory cortical microcircuits (Zempeltzi et al., 2020) and human prefrontal cortex for working memory (Finn et al., 2019), our method could be a useful tool to study the general layer-dependent feature in the cerebral cortex.

Finally, we performed the simulation of the multi-columnar model with layer 6 removed or the pathway from layer 6 to layer 4 blocked and confirmed the indispensable role of layer 6 for the concurrent case of opposite responses in layer 2/3 and layer 5. Layer 6 in the mouse's primary visual cortex was found to modulate the gain of sensory-evoked responses in upper layers, affecting their activity without altering their response characteristics, thereby acting as a crucial regulator in cortical visual processing (Olsen et al., 2012; Vélez-Fort and Margrie, 2012).

Building upon our findings, future research can delve deeper into the causes of the complex layer-dependent dynamics within neocortical columns and elucidate the underlying mechanisms driving their interactions. For example, bottom-up and top-down selective attention are thought to be integrated by a winner-take-all mechanism in spatial saliency maps (Koch and Ullman, 1985; Gan et al., 2023), so our layer-dependent approach can be helpful to model the computations on those maps. Such exploration may uncover new facets of neuronal activity, paving the way for more comprehensive models and understanding of the cortical micro-circuitry.

## Data Availability

The datasets presented in this study can be found in online repositories. The name of the repository can be found in the article.
